# Enhancing cooperativity of molecular J-aggregates by resonantly coupled dielectric metasurfaces

**DOI:** 10.1515/nanoph-2024-0117

**Published:** 2024-06-10

**Authors:** Marco Marangi, Yutao Wang, Mengfei Wu, Febiana Tjiptoharsono, Arseniy I. Kuznetsov, Giorgio Adamo, Cesare Soci

**Affiliations:** Centre for Disruptive Photonic Technologies, 54761 The Photonics Institute, Nanyang Technological University, Singapore 637371, Singapore; Division of Physics and Applied Physics, School of Physical and Mathematical Sciences, 54761 Nanyang Technological University, Singapore 637371, Singapore; Institute of Materials Research and Engineering, Agency for Science, Technology and Research (A*STAR), Singapore 138634, Singapore; Interdisciplinary Graduate School, Energy Research Institute @NTU (ERI@N), Nanyang Technological University, Singapore 637553, Singapore

**Keywords:** metasurfaces, J-aggregates, photoluminescence enhancement, superradiance, cooperativity

## Abstract

J-aggregates are supramolecular assemblies of dyes exhibiting strong absorption and fluorescence with narrow linewidths, as well as large optical nonlinearities, induced by the formation of largely delocalized molecular excitons. The degree of cooperativity achievable in J-aggregates ensembles, however, is limited by local disorder and thermally induced decoherence effects. A way to overcome these limitations and increase molecular exciton delocalization and coherence is to couple the ensemble of highly ordered molecular dipoles to a common electromagnetic mode in an optical resonator. In this work, we use dielectric metasurfaces to alter the radiative properties of coupled J-aggregate films and demonstrate a 5-fold Purcell enhancement of the luminesce intensity and narrowing of the emission directivity down to ∼300 mrad around the normal. These results highlight the potential of designer dielectric metasurfaces to foster the emergence of cooperative phenomena in excitonic systems, including optical nonlinearities and superradiance.

## 1 Introduction

**Figure 1:**
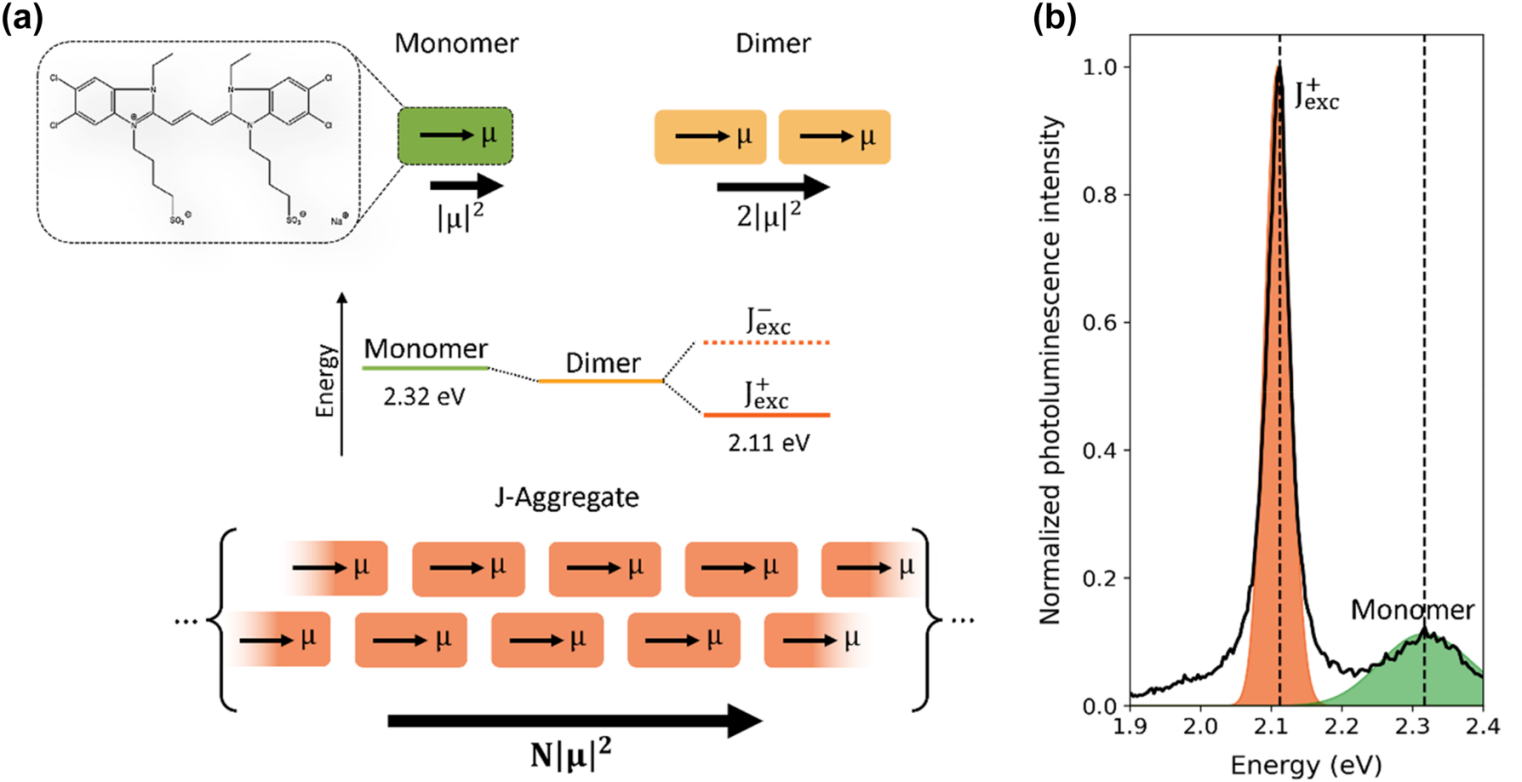
Dipole moments and photoluminescence of the TDBC monomer and J-aggregate. (a) Schematic configurations of a monomer (green box), dimer (yellow boxes) and J-aggregate stacking (orange boxes) of TDBC cyanine dye. *μ* is the dipole moment of individual monomeric units, and the bulk oscillator strength resulting from interacting dipole moments is shown below each structure (monomer, dipole, J-aggregate). (b) Experimental photoluminescence spectrum of a J-aggregate film at early stage of aggregation, deposited on quartz, showing a characteristic, weakly emissive monomeric band around ∼2.32 eV (green shaded curve) and a highly emissive, red-shifted J-aggregate exciton band around ∼2.11 eV (orange shaded curve).

J-aggregates are a class of molecular aggregates comprised of highly ordered supramolecular assemblies of dyes or other chromophores, where the formation of extended regions of coupled excitons with aligned transition dipole moments induces ensemble optical and electronic response (Figure 1(a)) [1], [2]. Typically, such systems are characterized by intense and narrow absorption and fluorescence peaks (Figure 1(b)) with large bathochromic shifts (i.e., red-shifts) and faster dynamics with respect to their monomeric constituent units, and size-enhanced third order non-linear optical susceptibility [3]. Because of the inherent alignment of the dipole moments, J-aggregates are an ideal system to observe cooperative behaviour in the form of superradiance (SR), a phenomenon originally described by Dicke in 1954 [4]. Superradiance occurs when an ensemble of *N* incoherent emitters coherently couple and form a macroscopic transition dipole moment, leading to luminescence emission rate and intensity that scale as *N* and *N* ^2^, respectively (Figure 1(a)). With recent advances in bottom-up aggregation of large ensembles of solid-state emitters and fabrication of optical cavities, superradiant nanophotonic devices have started to gain traction for applications in energy efficient and ultrafast emitters [5]–[8]. Nonetheless, stability, local disorder, and the presence of thermally induced decoherence effects, hinder the development of superradiant devices based on quantum dots [5] and J-aggregates [9].

A way to increase molecular exciton delocalization and coherence is to couple the ensemble of highly ordered molecular dipoles to a common electromagnetic mode in an optical resonator [10]. Alongside gratings, Fabry–Perot cavities and other resonant nanostructures [11]–[13], metasurfaces (two-dimensional planar arrangements of nanoscale optical resonators) have emerged as a versatile platform to achieve high confinement and manipulation of electromagnetic fields at the subwavelength scale. Both metallic and dielectric metasurfaces have been successfully employed to control amplitude, polarization and phase of reflected, transmitted or emitted light [13]–[18], [19]. Of particular relevance is the capability of light emitting metasurfaces to foster strong light–matter interaction and control the luminescence characteristics [20]–[23]. In this work, we design and fabricate dielectric metasurfaces and couple them with J-aggregates. We infer that aggregates coupled to the electromagnetic modes of the metasurfaces emit cooperatively, as revealed by the strong increase in intensity and decrease in angular distribution of the photoluminescence. We argue that this strategy may facilitate the onset of threshold-less coherent emission, and possibly the realization of energy-efficient superradiant devices operating at room temperature.

## 2 Dielectric metasurface with tunable electric and magnetic Mie resonances

**Figure 2:**
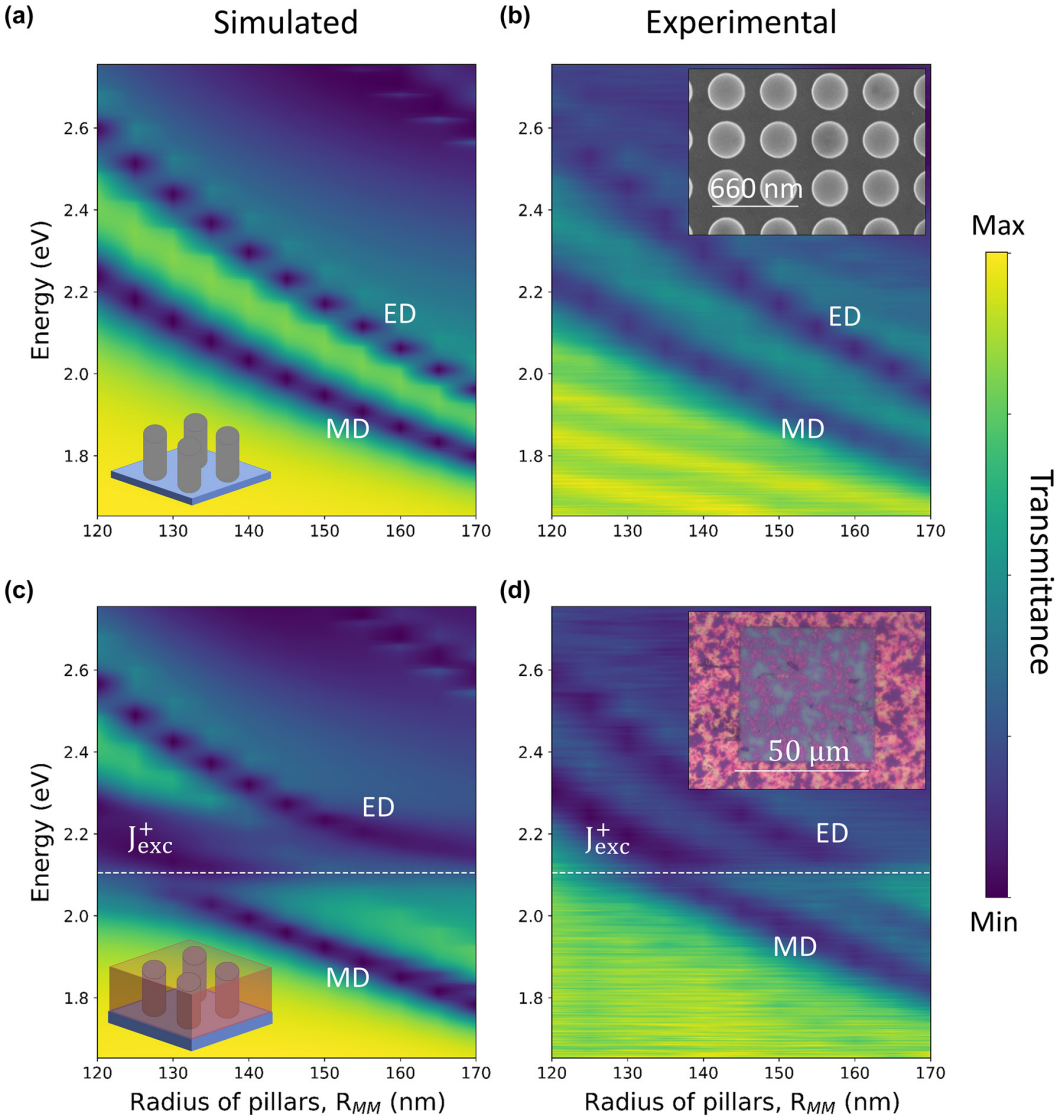
Coupling between the J-aggregate exciton and the metasurface optical modes (a) simulated and (b) experimental transmittance spectra of 200 nm thick TiO_2_ nanopillar metasurfaces as function of the nanopillars radius, *R*_*MM*_. The dark blue bands (dip in transmittance) correspond to the magnetic (MD) and electric (ED) dipole Mie resonances induced by localised displacement currents. (c) Simulated and (d) experimental transmittance spectra of the TiO_2_ metasurfaces coated with a ∼240 nm thin film of J-aggregate as function of the nanopillars radius, *R*_*MM*_. Both MD and ED bands show a slight red-shift induced by the refractive index of the J-aggregate film when they are far detuned from the excitonic resonance of the J-aggregate ( $J_{\text{exc}}^{+}$ ), and a discontinuity around ∼2.11 eV (white dashed line) induced by resonant coupling with the J-exciton.

Our metasurfaces consist of 50 μm × 50 μm arrays of titanium dioxide (TiO_2_) nanopillars on a quartz substrate, designed to support magnetic (MD) and electric dipole (ED) Mie resonances in the visible spectral range, induced by the displacement currents created in the nanopillars by the incident light [24]. TiO_2_ was chosen for its high refractive index and low losses around the wavelength of the TDBC J-aggregate exciton (∼2.11 eV). To find an optimal overlap between the metaurfaces resonances and the J-agregate excitons, we designed and fabricated metasurfaces with nanopillars of height *h* = 200 nm and radius, *R*_*MM*_, varying from 120 to 170 nm. All the TiO_2_ metasurfaces support ED and MD resonances, manifested by dips in transmission spectra that redshift from ∼2.6 eV to ∼2 eV (ED) and from ∼2.2 eV to ∼1.8 eV (MD) when the radius of the nanopillars increases, as predicted by full wave finite-difference time-domain (FDTD) electromagnetic simulations (Figure 2(a)) and confirmed by experiments (Figure 2(b)).

J-aggregate/metasurface coupled systems were obtained by spin-coating a ∼240 nm film of J-aggregate on top of the TiO_2_ metasurfaces. Due to the colloidal nature of the solution and the size of the aggregates, the resulting film is not entirely uniform across the nanopillar array (inset of Figure 2(d)). Despite the film inhomogeneity, FDTD simulations where J-aggregates are modelled as a continuous, 240 nm thick film covering the metasurface (Figure 2(c)), with effective refractive index adapted from reference [25] (the index was modelled as a Lorentzian function with *n*_real_ = 3.2 and *k*_imaginary_ = 2.6 at the exciton energy), are in very good agreement with the experimental spectra (Figure 2(d)). The transmission spectra of the J-aggregate/metasurface coupled systems show a slight red-shift of both the ED and MD Mie resonances when they are far detuned from the excitonic resonance of the aggregate, induced by the high refractive index of the J-aggregate film surrounding the resonators. On the other hand, when the Mie resonances of the metasurface and the excitonic resonances of the aggregate have a large spectral overlap, the coupling becomes stronger: this is revealed by the discontinuity of the MD and ED resonance energy as a function of the radius of the nanopillars at *R*_*MM*_ = 130 and *R*_*MM*_ = 160 nm, respectively (Figure 2(c) and (d)).

## 3 Photoluminescence enhancement in the coupled J-aggregate/metasurface system

**Figure 3:**
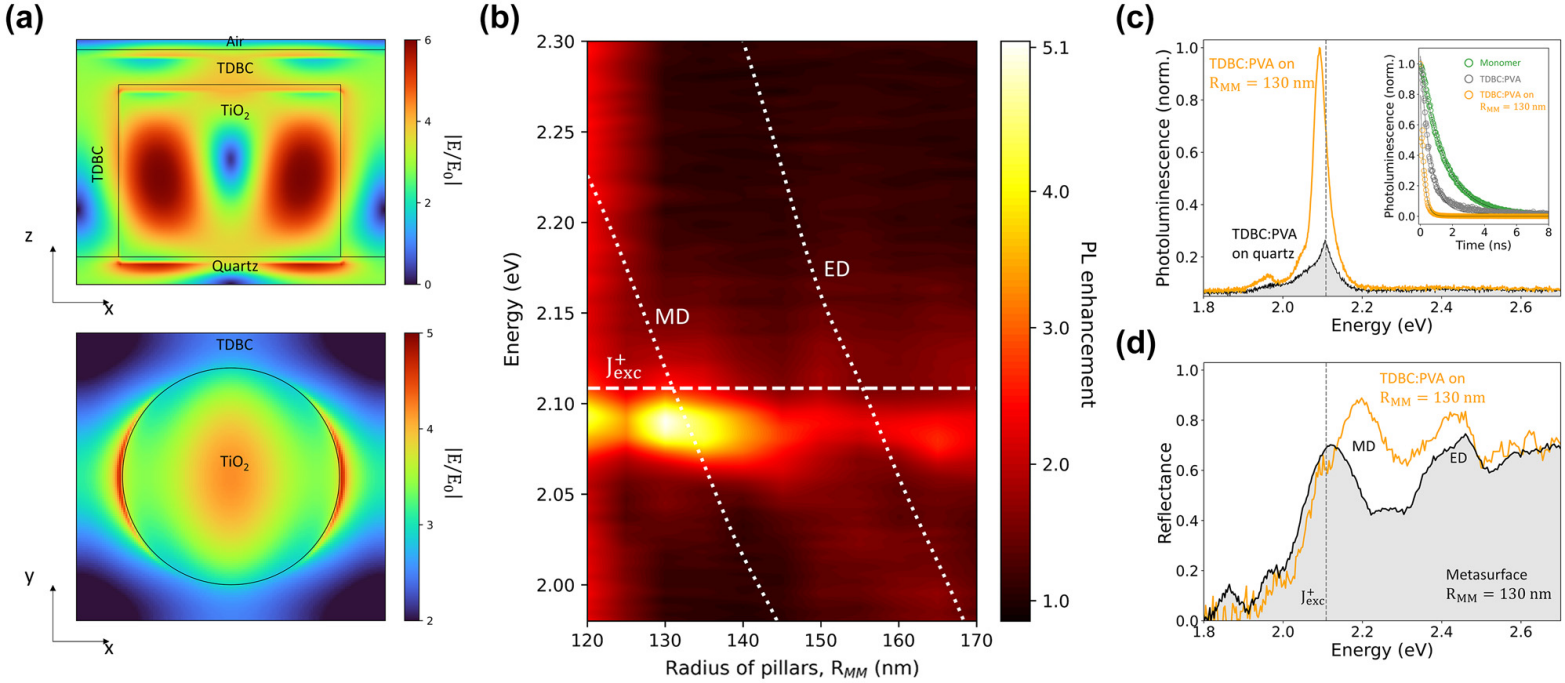
Photoluminescence emission enhancement in the J-aggregate/metasurface coupled system (a) near-field electric field intensity distribution in the vertical (top) and horizontal cross-section (bottom) of the resonant MD Mie photonic mode. (b) Photoluminescence enhancement factor, $\eta =\frac{PL_{J-agg+Meta}}{PL_{J-aggr}}$ , as a function of the nanopillar radius, *R*_*MM*_, showing strong enhancement when the J-aggregate exciton resonantly couples to MD and ED modes. The maximum (5-fold) enhancement is obtained for the MD mode of a nanopillar with radius *R*_*MM*_ = 130 nm. (c) Photoluminescence spectra of fully converted J-aggregate films deposited on bare quartz (gray shaded curve) and on the metasurface with maximum spectral overlap (MD), showing intensity enhancement and energy redshift. Inset: Time resolved PL for monomer (green circles), TDBC (grey circles) and TDBC on the *R*_*MM*_ = 130 nm metasurface (yellow circles). (d) Reflectance spectrum of the J-aggregate/metasurface coupled system (black curve) showing the interaction between the resonant photonic mode (MD) and the J-exciton (gray dashed line), which broadens and shifts the MD resonance (the shaded gray area shows the reflectance of the bare TiO_2_ metasurface).

Coupling between J-aggregates and metasurfaces increases the local density of optical states (LDOS) in the vicinity of the nanopillars. Figure 3(a) shows the distribution of the electric field amplitude of the MD resonance in a 130 nm radius nanopillar in the vertical (*xz* plane, top panel) and horizontal (*xy* plane, bottom panel) cross-sections of the unit cell, at the emission energy of the J-exciton. Both views show that the MD mode extends into the TDBC layer, enhancing the electromagnetic field within the active medium.

The coupling strength between the J-exciton and the photonic modes of the metasurface determines the extent of photoluminescence (PL) enhancement due to the Purcell effect [26]. The colormap of Figure 3(b) shows the PL enhancement as a function of pillar radius and photon energy. The two distinct luminescence bands, red-shifted from the emission of the uncoupled J-aggregate, can be attributed to the exciton coupled to the MD mode (from *R*_*MM*_ = 120 nm to *R*_*MM*_ = 145 nm) and to the ED mode (from *R*_*MM*_ = 150 nm to *R*_*MM*_ = 170 nm), respectively. In both cases, the maximum PL intensity red-shifts with increasing radius of the nanopillar. The optimal mode overlap with the excitonic peak of TDBC, yielding a maximum 5-fold enhancement of luminescence, occurs for the MD resonance of nanopillars with *R*_*MM*_ = 130 nm. The corresponding PL spectrum is shown in Figure 3(c) (yellow line), together with the reference spectrum of the J-aggregate film on unpatterned quartz (grey shaded curve). The additional low-energy shoulders appearing in fully converted films may be attributed to inhomogeneous aggregates formed at later stages. Besides the enhancement, the PL peak of the coupled J-exciton slightly red-shifts compared to the reference spectrum, which indicates an increase in conjugation length induced by the resonantly coupled metasurface. Furthermore, the red shift may point to the fact that the coupled system is approaching the strong-coupling regime, as also hinted by the discontinuity of the MD and ED resonance energy seen in Figure 2(c) and (d). The increase in the exciton coherence length can be estimated from the photoluminescence lifetime, as $N_{\text{coherent}}≅\frac{\tau_{\text{monomer}}}{\tau_{J}}$ [26]. Time resolved PL measurements (inset of Figure 3(c)) for monomers, TDBC and TDBC on the *R*_*MM*_ = 130 nm metasurface give *τ*_monomer_ ≅ 1.48 ns, *τ*_*J*_ ≅ 0.61 ns and *τ*_*J on MM*_ ≅ 0.22 ns, corresponding to *N*_coherent_ of approximately 2 and 7, respectively. Redistribution of the exciton transition energy above and below that of the uncoupled J-exciton is also seen in the reflectance spectrum of the coupled J-aggregate shown in Figure 3(d), where the MD peak (yellow curve) appears to be blue-shifted compared to the reference spectrum (grey shaded curve).

## 4 Directional emission from the J-aggregate/metasurface coupled system

**Figure 4:**
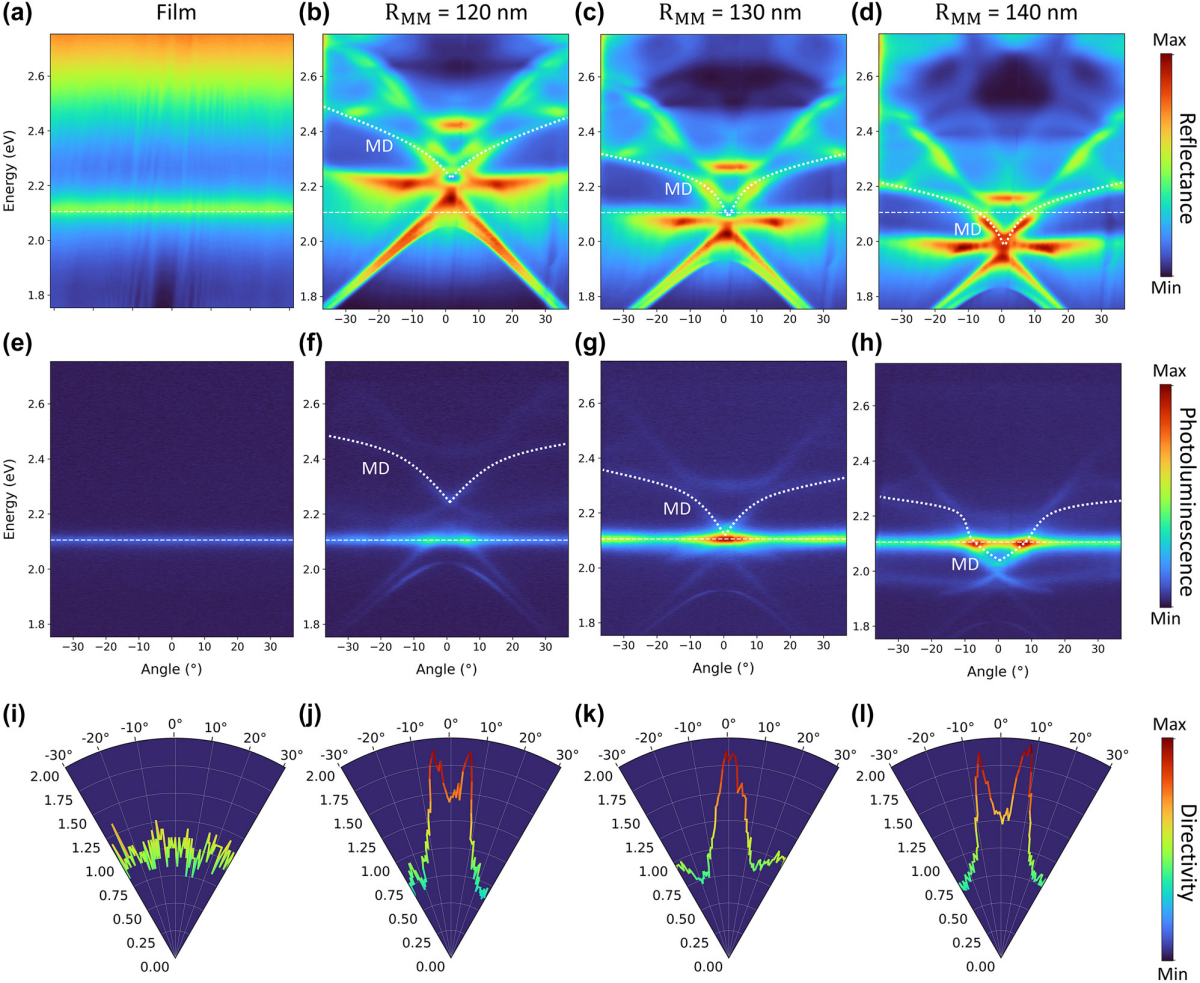
Directional photoluminescence emission of the J-aggregate/metasurface coupled system. Angle-resolved reflectance spectra of J-aggregate films deposited on (a) unpatterned quartz and nanopillars metasurfaces with of radius (b) *R*_*MM*_ = 120 nm, (c) *R*_*MM*_ = 130 nm, (d) *R*_*MM*_ = 140 nm. (e–h) Corresponding angle-resolved photoluminescence spectra for the same cases. Both reflectance and photoluminescence maps show a complex photonic bandstructure, which redshifts with increasing *R*_*MM*_. Coupling between the excitonic band of the J-aggregate (white dashed lines) and the MD mode of the metasurfaces (white dashed lines) is revealed by the change in curvature of MD photonic bands around the energy of the exciton (∼2.11 eV) and the PL enhancement where the excitonic and photonic bands cross (f–h). (i–l) Directivity of the photoluminescence highlighting the change from isotropic exciton emission (i) to directional emission after coupling to the MD mode of the metasurfaces (j–l). Maximally directional emission along the normal (*k*_*x*_ = 0) is obtained when the cusp of the MD mode overlaps with the J-exciton (g), corresponding to a narrowing of the emission directivity to ∼300 mrad around the normal.

To obtain further insights on the influence of the metasurface on the radiative properties of J-aggregates, we measured the angle (momentum) dependent energy dispersion of reflectance (Figure 4(a–d)) and photoluminescence (Figure 4(e–h)) by back focal plane imaging, comparing four cases: the J-aggregate film on an unpatterned substrate and J-aggregate films deposited on metasurfaces with MD resonances blue-detuned (*R*_*MM*_ = 120 nm), overlapping (*R*_*MM*_ = 130 nm) and red-detuned (*R*_*MM*_ = 140 nm) with respect to the J-exciton energy (∼2.11 eV). As expected, reflection and PL emission of the J-aggregate film on unpatterned quartz show a dispersion-less sharp peak around the exciton energy (Figure 4(a–e), respectively), and their angular distribution is isotropic (Figure 4(i)). When the J-aggregate is coupled to dielectric metasurfaces with increasing *R*_*MM*_, the resonant photonic modes of the metasurface induce a slight red-shift of the PL emission (Figure 3(b)), and the angular distribution becomes strongly anisotropic (Figures 4(j–l)). The largest directionality of ∼300 mrad around the normal direction is obtained for the MD array with *R*_*MM*_ = 130 nm, for which the dispersion of the cavity mode has an inflection point with the cusp matching the energy of the J-exciton. This increases the density of optical states around the normal direction at the energy of the J-aggregate excitonic peak. Directional emission of the J-aggregate/metasurface system may be seen as a further indication of the enhanced spatial coherence of emitters coupled with a common photonic mode of the metasurface, whereby the coupling is expected to foster cooperativity by reducing the local intra-aggregate disorder [10].

## 5 Summary and conclusions

In summary, we successfully demonstrated coupling of resonant dielectric metasurfaces to self-assembled molecular J-aggregates, and suggested the use of this platform to promote the emergence of cooperativity in molecular J-aggregates by subjecting them to a common photonic mode of the metasurface. Through both experiments and numerical simulations, we have shown that the increase of local density of optical states induced by resonant dielectric metasurfaces that support (single particle) magnetic and electric dipole modes leads to considerable enhancement of the luminescence intensity (up to 5-fold). We have also shown that coupling J-excitons to the photonic bands of the metasurfaces imparts directionality to the light emitted along the normal direction. While this first demonstration probed the ensemble-averaged behaviour of small groups of J-aggregates on the metasurface, we envisage that non-local photonic modes (e.g. bound states in the continuum or lattice modes) could be used to extend cooperativity of a larger number of aggregates, as a route to phase the emitting dipoles and possibly enhance superradiance. Overall, we believe that dielectric metasurfaces are an ideal platform to engineer ad-hoc photonic modes that strongly couple to the naturally aligned transition dipole moments of molecular excitons to take advantage of their high optical nonlinearities and fast response time, which may ultimately lead to the development of power-efficient, superradiant light-emitting devices.

## 6 Methods

### 6.1 Metasurface design

The design of the dielectric metasurface was optimized via finite-difference time-domain (FDTD) numerical simulations by using the commercial software Ansys Lumerical FDTD. The simulated unit cell consisted of a TiO_2_ cylinder of 200 nm in thickness with variable radius and period (*x*_period_ = 2*R* + 100 nm, *y*_period_ = 2*R* + 75 nm), on top of a quartz substrate and surrounded by air. In the active simulations we assumed a 240 nm thick TDBC film fully covering the metasurface. In all simulations the rectangular unit cell was surrounded by periodic boundary conditions, and was excited by a *x*-polarized plane wave propagating from the bottom of the substrate to the top. The transmittance was detected by a monitor placed at the top of the metasurface and was computed for different radii of the nanopillars. The electric field distributions were calculated by placing a monitor along the *x*–*z* plane and *x*–*y* plane.

### 6.2 Metasurface fabrication

200 nm-thick TiO_2_ was deposited on quartz by ion-beam sputtering with a collimated Ar^+^ beam (Oxford Instruments Optofab 3,000) at a deposition rate of 0.2 Å/s. In addition, 2 sccm of O_2_ was flown into the chamber at 4.5 × 10^−4^ Torr to adjust the stoichiometry of the sputtered TiO_2_. We then deposited 30 nm of Cr on TiO_2_ by electron-beam evaporation (Angstrom EvoVac) at a rate of 1 Å/s and chamber pressure of 4 × 10^−6^ Torr. Hydrogen silsesquioxane (HSQ, Dow Corning XR-1541-006) resist was spin coated on the sample at 5,000 rpm for 60 s and baked at 180 °C for 3 min. Electron-beam lithography was carried out with an exposure dose of ∼10 mC/cm^2^ (Elionix ELS-7000). The resist was developed by a salty solution (1 wt% NaOH and 4 wt% NaCl in deionized water) for 2 min, followed by a rinse in deionized water. Cr was etched first with inductively coupled plasma reactive ion etching (ICP-RIE, Oxford Plasmalab System 100), using 19 sccm of Cl_2_ and 2 sccm of O_2_ at 10 mTorr. TiO_2_ was etched, using the Cr as a hard mark, with 25 sccm of CHF_3_ at 25 mTorr. Finally, the Cr was removed by immersion in liquid etchant for 4 min. The sample was rinsed by deionized water and isopropanol and blow-dried with N_2_.

### 6.3 Processing and deposition of TDBC J-aggregates

The cyanine dye 5,6-Dichloro-2-[[5,6-dichloro-1-ethyl-3-(4-sulfobutyl)-benzimidazol-2-ylidene]-propenyl]-1-ethyl-3-(4-sulfobutyl)-benzimidazolium hydroxide, inner salt, sodium salt J-aggregate (TDBC) was purchased from Biosynth Carbosynth (CAS: 18462-64-1) and was further purified with methanol following the procedure described by Barotov et al. [27]. The purified monomer solution was then mixed with de-ionized water with a TDBC monomer concentration of $2.5\frac{\text{mg}}{\text{mL}}$ . The aggregate was left to grow in the dark at ambient temperature for 3 days. Concurrently, $65\frac{\text{mg}}{\text{mL}}$ of Poly(Vinyl Alcohol) (PVA), acquired from Sigma Aldrich (CAS: 9002-89-5), were further polymerized in water for 4 h at 140 °C under constant stirring. A volume of 70 μL of grown TDBC was then thoroughly mixed with 6 μL of polymerized PVA. Static spincoating was employed to deposit 70 μL of this solution at 2300 rpm for 60 s on top of the patterned TiO_2_ metasurface. The sample was then further dried with N_2_.

### 6.4 Angle-resolved spectroscopy

Angle-resolved reflectance (ARR) and photoluminescence (ARPL) measurements were performed with a custom built microspectrometer setup consisting of an inverted optical microscope (Nikon Ti-u, 50× objective, NA = 0.6), a spectrograph (Andor SR-303i with a 300 lines/mm grating), and a charged-coupled detector (CCD, Andor iDus 420). A series of lenses along the beam path between the microscope and the spectrograph projects the back focal plane (BFP) of the collection objective on the slit of the spectrograph, allowing the collection of angular information within bounds defined by $\frac{k_{x}}{k_{0}}=\text{NA}=0.6$ . The sample was excited via a halogen lamp for ARR measurements, while a 405 nm continuous wave laser was used for ARPL measurements.
